# Prognostic Value of the Systemic Immune-Inflammation Index and Prognostic Nutritional Index in Patients With Medulloblastoma Undergoing Surgical Resection

**DOI:** 10.3389/fnut.2021.754958

**Published:** 2021-12-17

**Authors:** Sihan Zhu, Zhuqing Cheng, Yuanjun Hu, Zhenghe Chen, Ji Zhang, Chao Ke, Qunying Yang, Fuhua Lin, Yinsheng Chen, Jian Wang

**Affiliations:** Department of Neurosurgery and Neuro-Oncology, State Key Laboratory of Oncology in South China, Collaborative Innovation Center for Cancer Medicine, Sun Yat-sen University Cancer Center, Guangzhou, China

**Keywords:** medulloblastoma, systemic immune-inflammation index, prognostic nutritional, overall survival, ALBI (albumin-bilirubin) score

## Abstract

**Background:** The progression and metastasis of cancers are associated with systematic immune inflammation and nutritional dysfunction. The systemic immune-inflammation index and prognostic nutritional index (PNI) have shown a prognostic impact in several malignancies. Therefore, our study aimed to evaluate immune inflammation and nutritional index prognostic significance in patients with medulloblastoma (MB).

**Methods:** We retrospectively analyzed 111 patients with MB between 2001 and 2021 at our institution. The optimal cutoff values for systemic immune-inflammation index (SII), neutrophil/lymphocyte ratio (NLR), monocyte/lymphocyte counts ration (MLR), and PNI were evaluated with receiver operating characteristic (ROC) curve analysis. Clinical characteristics and SII, NLR, MLR, and PNI were tested with the Pearson's chi-squared test. The Kaplan–Meier survival curves and the Cox proportional hazards model were used to evaluate the effects of immune inflammation and nutritional index on overall survival (OS).

**Results:** Receiver operating characteristic curve analysis determined the optimal SII, NLR, MLR, and PNI cutoff values of 2,278, 14.83, 0.219, and 56.5 that significantly interacts with OS and divided the patients into two groups. Comparative survival analysis exhibited that the high-SII cohort had significantly shorter OS (*p* = 0.0048) than the low-SII cohort. For the univariate analysis, the results revealed that preoperative hydrocephalus (*p* = 0.01), SII (*p* = 0.006), albumin–bilirubin score (ALBI) (*p* = 0.04), and coSII–PNI were predictors of OS. In the multivariate analysis, preoperative hydrocephalus (*p* < 0.001), ALBI (*p* = 0.010), SII (*p* < 0.001), and coSII–PNI as independent prognostic factors were significantly correlated with OS.

**Conclusion:** The preoperative SII, ALBI, and coSII–PNI serve as robust prognostic biomarkers for patients with MB undergoing surgical resection.

## Introduction

Medulloblastoma (MB) is the most common malignant pediatric brain tumor, accounting for about 20% of central nervous system tumors of children. The current standard treatment includes surgical intervention, craniospinal irradiation, and adjuvant chemotherapy (1). The 5-year overall survival (OS) was 85% for patients with the average risk and 70% for those with the high risk (2). Sadly, this treatment has a high morbidity and mortality rate, and even those children cured are left with lifelong sequelae, including neurological, cognitive, and endocrine disorders (3). Therefore, reliable biomarkers were strongly demanded to guide treatment decisions and optimize patient outcomes.

A growing body of evidence suggested that the interaction of inflammation and the immune system with cancer cells plays a crucial role in the genesis, proliferation, progression, and metastasis of the tumors (4). Meanwhile, inflammatory pathways are considered critical targets for improving therapeutic efficacy (5). Therefore, given the relationship between inflammation and tumor development, it is of great significance to explore the application of inflammatory markers in the tumor therapy. Nutrition is essential for proper growth and development and is a critical component in optimizing clinical outcomes. Malnutrition is an adverse prognostic factor in children with cancer, and its prevalence is highly variable (6).

For the past few years, emerged novel prognostic predictive index that has been verified in many tumors. Some simple biochemical indicators can reflect systemic inflammatory response and nutritional status. Systemic immune-inflammation index (SII) was calculated by counting platelets, neutrophils, and lymphocytes in the peripheral blood. The prognostic nutritional index (PNI) was determined by serum albumin concentration and peripheral blood lymphocyte count. These two systemic immune and nutritional indicators have been shown to play a predictive role in many types of cancer (7).

Although there is compelling underlying evidence in many types of cancer, the prognostic utility of SII and PNI in patients with MB has, to date, surprisingly, not been carefully studied. This study focused on the prognostic role of immunoinflammatory and prognostic nutrition indicators in patients with MB.

## Patients and Methods

### Patients Section

We retrospectively analyzed the medical data of newly diagnosed 111 patients with MB at our hospital from January 2001 to March 2021. Participants were in this study if they (1) were pathologically diagnosed with MB, (2) without any antitumor treatment, (3) with complete medical follow-up data and laboratory results. The participants were excluded (1) with other primary malignant tumors found during the treatment, (2) with liver and kidney dysfunction that occurred during treatment, (3) using nonsteroidal anti-inflammatory drugs.

### Ethics, Consent, and Permissions

We conducted the study per the principles of the Declaration of Helsinki and the Rules of Good Clinical Practice. Furthermore, the study design was approved by the Institutional Ethical Committee review board of Sun Yat-sen University Cancer Center (B2020-360-01) before the acquisition of any patient information. According to our institutional standards, all the patients provided a written informed consent, either by themselves or by a legally authorized representative, to collect and analyze blood samples, pathology samples, and publish their results before starting treatment.

### Data Collection and Definition

The clinical parameters of patients such as gender, age, tumor size, tumor location, the Karnofsky Performance Scale (KPS), preoperative hydrocephalus, preoperative headache status, preoperative vomiting status, preoperative ataxia status, duration of symptoms, ventricular drainage status, surgery extent, metastasis status, recurrence status, postoperative radiotherapy, postoperative chemotherapy status, and laboratory data were collected from the medical data. We collected all laboratory parameters during routine tests before cancer diagnostic intervention. The ALBI grades were calculated using the following equation: 0.66 × log_10_ bilirubin level−0.085 × albumin level. The ALBI grades were stratified into grade one ( ≤ -2.60) and grade two (−2.59 to −1.39). The PALBI grades were calculated using the following equation: 2.02 × log_10_ bilirubin level−0.37 × (log_10_ bilirubin level)−0.04 × albumin level−3.48 × log_10_ platelet count + 1.01 × (log_10_ PLT) (8). The PALBI grades were stratified into grade one ( ≤ -2.53), grade two (−2.52 to −2.09), and grade three (>-2.09) (8). The definitions of SII, NLR, MLR, and PNI were shown as follows: PNI = albumin (g/L) + 5 × total lymphocyte counts (10^9^/L); SII = platelet × neutrophil/lymphocyte counts; NLR = neutrophil/lymphocyte counts; MLR = monocyte/lymphocyte counts, and PNI = albumin (g/L) + 5 × total lymphocyte counts (10^9^/L). According to the existing literature reports, we will give those with low SII and high PNI score 2; those with high SII and high PNI or low SII and low PNI score 1, and those with high SII and low PNI score 0 (5). The extent of resection was recorded as gross total resection (GTR), subtotal resection (STR), and biopsy. We determined the extent of excision based on an enhanced MRI scan of the head taken within 72 h postoperatively and surgical records.

### Follow-Up

All the postoperative patients will be regularly followed-up by full-time staffs. Follow-up work have continued until death or April 2021. OS is considered to be the time between surgery and tumor-related death or last contact.

### Statistical Analysis

Software of the SPSS version 23.0 (IBM Corp, Armonk, NY) and the GraphPad Prism version 8.0 (La Jolla, CA, USA) was utilized to perform statistical analysis. Median value and range were used for the quantitative variables, while the categorical variables were described as frequency and percentage. We used the chi-squared or the Fisher's exact tests to compare groups. The receiver operating characteristic (ROC) curve analysis was utilized to get the optimal cutoff values of predictors by the highest Youden's index. Survival analyses were performed using the Kaplan–Meier survival curves. This study utilized Bonferroni correction and related *p*-values for comparisons between three or more groups. The Cox proportional hazards regression model investigated the univariate and multivariate analyses. Hazard ratios (HRs) and 95% CIs were utilized to evaluate relative risk. A two-tailed *p* < 0.05 was suggested remarkably significant.

## Results

### Patient Demographics

We identified diagnosed 311 patients with MB, but 200 patients were excluded from the analysis because they only received radiotherapy or chemotherapy in our institution without surgical treatment, leaving 111 patients eligible for the following analysis. Patients with MB undergoing surgical resection and disease characteristics for the whole study cohort are given in Table 1. The median age was 10 (range: 1–48) with male gender (63.9%) and children with age <18 years (76.5%) dominancy. The median KPS was 80 (range: 50–100). The preoperative headache, vomiting, and ataxia rates at presentation were 69.3, 64.8, and 52.2%, separately. Tumor size, metastasis status, ALBI, and PALBI are also shown in Table 1.

**Table 1:** Baseline patient and disease characteristics.

		**SII**			**NLR**			**MLR**			**PNI**		
		**Low**	**High**		**Low**	**High**		**Low**	**High**		**Low**	**High**	
**Variables**	**Cases**	**(102)**	**(9)**	***P***	**(105)**	**(6)**	***P***	**(57)**	**(54)**	***P***	**(64)**	**(47)**	***P***
Gender				0.58			0.88			0.03			0.43
Male	71	66	5		67	4		31	40		39	32	
Female	40	36	4		38	2		26	14		25	15	
Age(years)				0.46			0.68			0.01			0.36
<18	85	79	6		80	5		49	36		47	38	
≥18	26	23	3		25	1		8	18		17	9	
Tumor size				0.64			0.80			0.54			<0.01
≤ 4 cm	32	30	2		30	2		15	17		27	5	
>4 cm	79	72	7		75	4		42	37		37	42	
Tumor location				0.60			0.03			0.07			0.13
Subtentorial	108	99	9		103	5		57	51		61	47	
Subtentorial-supracerebellar	3	3	0		2	1		0	3		3	0	
KPS				0.50			0.96			0.16			0.71
≤ 70	38	34	4		36	2		16	22		21	17	
>70	73	68	5		69	4		41	32		43	30	
Preoperative hydrocephalus				0.28			0.49			0.54			0.53
Yes	79	74	5		74	5		42	37		47	32	
No	32	28	4		31	1		15	17		17	15	
Preoperative headache				0.18			0.09			0.002			0.055
Yes	77	69	8		71	6		32	45		49	28	
No	34	33	1		34	0		25	9		15	19	
Preoperative vomiting				0.90			0.92			0.99			0.84
Yes	72	66	6		68	4		37	35		42	30	
No	39	36	3		37	2		20	19		22	17	
Preoperative ataxia				0.83			0.91			0.93			0.86
Yes	58	53	5		55	3		30	28		33	25	
No	53	49	4		50	3		27	26		31	22	
Duration of symptoms				0.05			0.12			0.86			0.32
≤ 3 months	81	72	9		75	6		42	39		49	32	
>3 months	30	30	9		30	0		15	15		15	15	
Metastasis				0.45			0.80			0.07			0.07
Yes	23	22	1		22	1		8	15		17	6	
No	88	80	8		83	5		49	39		47	41	
ALBI				0.41			0.51			0.75			0.44
Grade 1	104	95	9		98	6		53	51		59	45	
Grade 2	7	7	0		7	0		4	3		5	2	
PALBI				0.14			0.83			0.10			0.37
Grade 1	83	75	8		78	5		46	37		48	35	
Grade 2	24	24	0		23	1		8	16		15	9	
Grade 3	4	3	1		4	0		3	1		1	3	
Mean OS time (month)		45.1	36.3		44.7	39.1		35.9	32.5		33.1	52.8	

*SII, systemic immune-inflammation index; NLR, neutrophil–lymphocyte ratio; MLR, monocyte–lymphocyte ratio; PNI, prognostic nutritional index*.

### Optimal Cutoff Value for SII, NLR, MLR, and PNI

**Figure 1:**
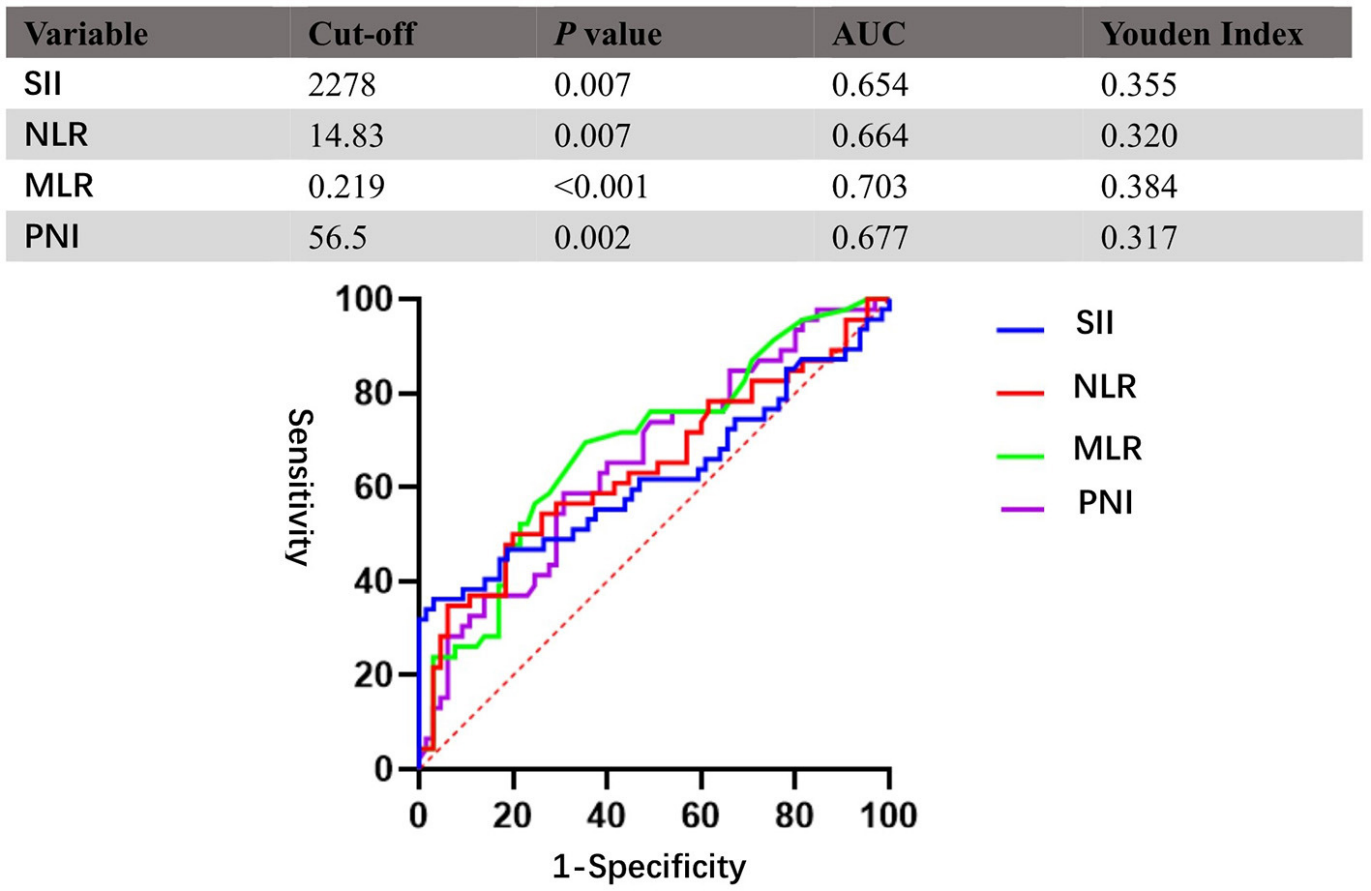
Receiver operating characteristic curves (ROCs) for pretreatment SII, NLR, MLR, and PNI based on OS. SII, systemic immune-inflammation index; NLR, neutrophil–lymphocyte ratio; MLR, monocyte–lymphocyte ratio; PNI, prognostic nutritional index.

This study applied ROC curve analysis as a more objective method to find an optimal cut-off for the possible connection between SII, NLR, MLR, PNI, and OS rather than bias-prone mean/median values. The optimal cut-off of SII, NLR, MLR, and PNI was 2,278, 14.83, 0.219, and 56.5, respectively. Details of the results are shown in Figure 1.

According to the aforementioned cut-off value, patients were divided into two groups: low-SII (<2,278, *n* = 102); high-SII (≥ 2,278, *n* = 9); low-NLR (<14.83, *n* = 105); high-NLR (≥ 14.83, *n* = 6); low-MLR (<0.219, *n* = 57); high-MLR (≥ 0.219, *n* = 54); low-PNI (<56.5, *n* = 64); and high-PNI (≥ 56.5, *n* = 47). Evaluation of baseline demographics (Table 1) and treatment characteristics (Table 2) per conveyance SII, NLR, MLR, and PNI groups revealed no meaningful differences between the two cohorts, except for tumor location (*p* = 0.03) in the NLR group. Difference in gender (*p* = 0.03), age (*p* = 0.01), preoperative headache (*p* = 0.002) in the MLR group and tumor size (*p* < 0.01) in the PNI group is also found significant.

**Table 2:** Treatment characteristics and clinical outcomes.

		**SII**			**NLR**			**MLR**			**PNI**		
		**Low**	**High**		**Low**	**High**		**Low**	**High**		**Low**	**High**	
**Variables**	**Cases**	**(102)**	**(9)**	***P***	**(105)**	**(6)**	***P***	**(57)**	**(54)**	***P***	**(64)**	**(47)**	***P***
Postoperative drainage				0.15			0.08			0.53			0.08
Yes	75	67	8		69	6		37	38		39	36	
No	36	35	1		36	0		20	16		25	11	
Recurrence				0.78			0.13			0.96			0.10
Yes	29	2	6		29	0		15	14		13	16	
No	82	75	7		76	7		42	40		51	31	
Surgical extent				0.84			0.78			0.29			0.42
GTR	79	73	6		74	5		40	39		46	33	
STR	30	27	3		29	1		17	13		16	14	
Biopsy	2	0	2		2	0		0	2				
Postoperative radiotherapy				0.53			0.16			0.31			0.59
Yes	67	62	5		65	2		37	30		40	27	
No	44	40	4		40	4		20	24		24	20	
Postoperative chemotherapy				0.53			0.73			0.84			0.90
Yes	63	57	6		60	3		33	30		36	27	
No	48	45	3		45	3		24	24		28	20	

*SII, systemic immune-inflammation index; NLR, neutrophil–lymphocyte ratio; MLR, monocyte–lymphocyte ratio; PNI, prognostic nutritional index; GTR, gross total resection; STR, subtotal resection*.

### Association of SII, NLR, MLR, and PNI With Survival Outcomes

**Figure 2:**
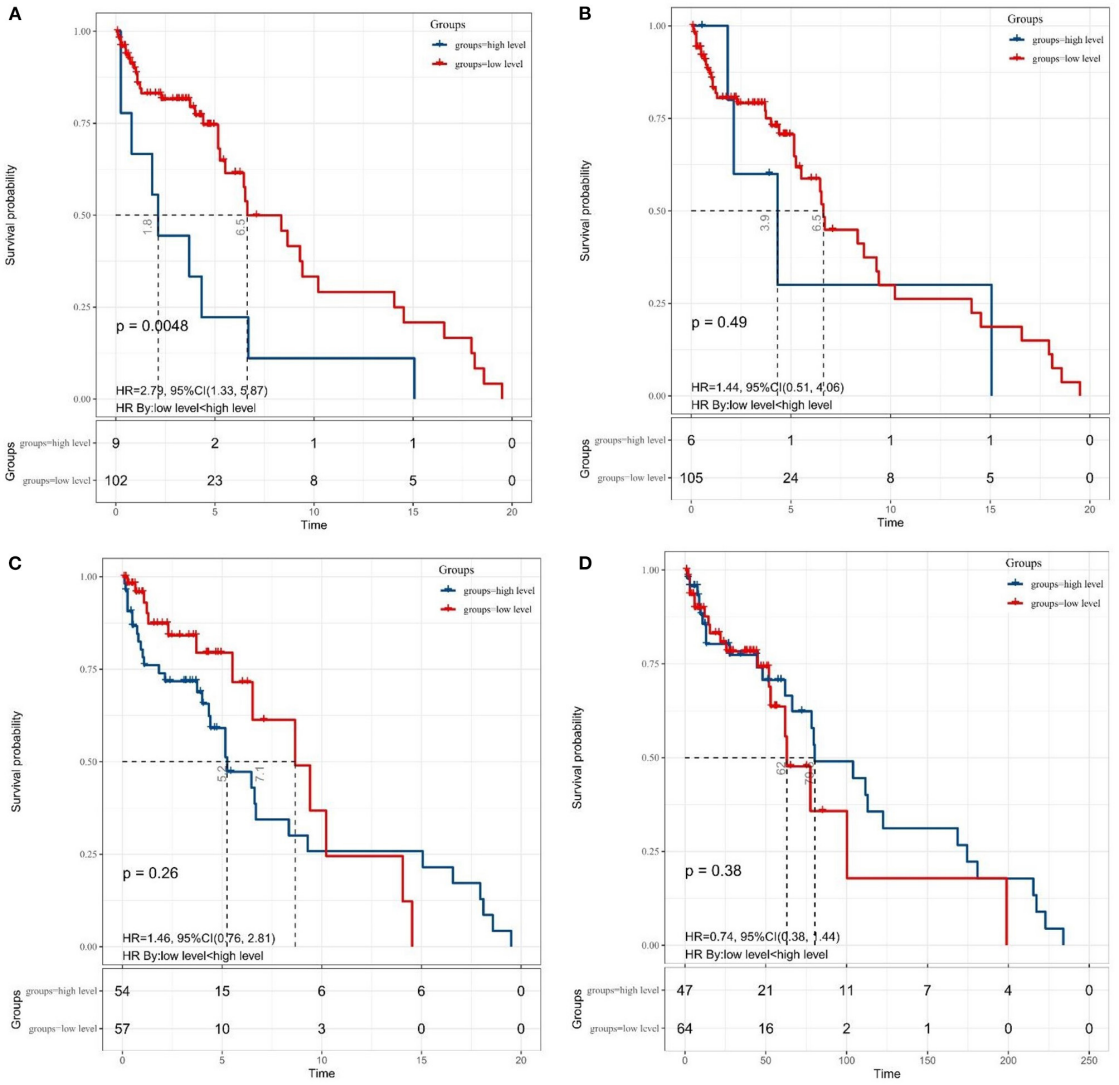
The Kaplan–Meier survival curves for OS according to **(A)** SII, **(B)** NLR, **(C)** MLR, and **(D)** PNI in patients with medulloblastoma. SII, systemic immune-inflammation index; NLR, neutrophil–lymphocyte ratio; MLR, monocyte–lymphocyte ratio; PNI, prognostic nutritional index.

**Figure 3:**
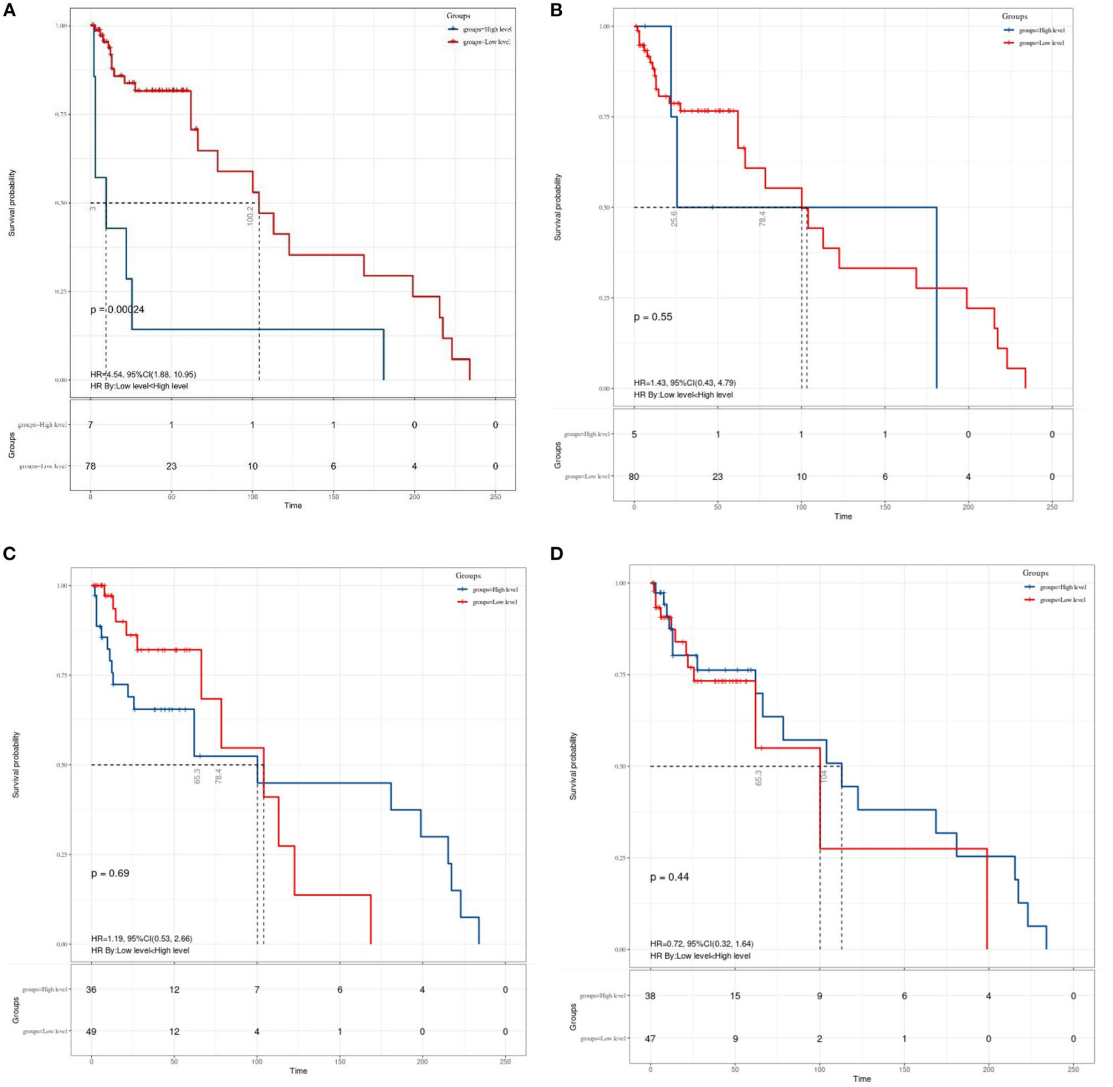
The Kaplan–Meier survival curves for OS according to **(A)** SII, **(B)** NLR, **(C)** MLR, and **(D)** PNI in patients aged ≤ 18 years with medulloblastoma. SII, systemic immune-inflammation index; NLR, neutrophil–lymphocyte ratio; MLR, monocyte–lymphocyte ratio; PNI, prognostic nutritional index.

Granting the endpoints of this study, we compared the outcomes of patients allocated to the low level and high level of SII, NLR, MLR, and PNI groups in terms of OS. As shown in Figure 2, results of the comparative analyses paraded that patients with MB in a high level of SII had significantly inferior OS (*p* = 0.0048) than patients in a low level of SII group. However, we did not find any significant difference in OS between low and high levels of patients in NLR (*p* = 0.49), MLR (*p* = 0.26), and PNI (*p* = 0.38) groups. Since MBs tend to occur in people younger than 18 years of age, we also made a subgroup analysis of the aforementioned immune inflammation and prognostic nutrition indexes (PNIs). Same as previous results (Figure 3), a high level of SII had significantly inferior OS (*P* = 0.00024) than patients in a low level of SII group in patients with MB aged ≤ 18. Unfortunately, we have not found any significant difference in OS between low and high levels of patients in NLR (*p* = 0.55), MLR (*p* = 0.69), and PNI (*p* = 0.44) groups.

### Univariate and Multivariate Survival Analyses

The univariate and multivariate survival analyses are shown in Table 3. On the univariate analysis, preoperative hydrocephalus (*p* = 0.01), SII (*p* = 0.006), and ALBI (*p* = 0.04) were significantly associated with OS. Patients in the high-level SII group had an increase in death risk compared with patients in the low SII level group. Besides, the death risks of patients suffering preoperative hydrocephalus increased a lot. A high-preoperative grade of AIBL, meaning worse hepatic function, increases the risk of death in patients with MB.

**Table 3:** The univariate and multivariate analyses of overall survival in patients with medulloblastoma.

	**Univariate analysis**		**Multivariate analysis**	
**Variables**	**HR (95% CI)**	***P***	**HR (95% CI)**	***P***
Gender		0.67		-
Male	Reference		NA	
Female	1.141 (0.617–2.109)		NA	
Age(years)		0.10		-
<18	Reference		NA	
≥18	1.703 (0.888–3.262)		NA	
Tumor size		0.28		-
≤ 4 cm	Reference		NA	
>4 cm	0.700 (0.366–1.340)		NA	
Tumor location		0.13		-
Subtentorial	Reference		NA	
Subtentorial-supracerebellar	2.975 (0.701–12.62)		NA	
KPS		0.66		-
≤ 70	Reference		NA	
>70	2.975 (0.701–12.62)		NA	
Preoperative hydrocephalus		0.01		<0.001
Yes	Reference		Reference	
No	0.870 (0.267–0.980)		0.047 (0.012–0.185)	
Preoperative headache		0.52		-
Yes	Reference			NA	
No	0.809 (0.420–1.559)		NA	
Preoperative vomiting		0.43		-
Yes	Reference		NA	
No	1.280 (0.690–2.375)		NA	
Preoperative ataxia		0.82		-
Yes	Reference		NA	
No	0.934 (0.515–1.694)		NA	
Duration of symptoms		0.77		-
≤ 3 months	Reference		NA	
>3 months	0.934 (0.515–1.694)		NA	
Metastasis		0.68		-
Yes	Reference			NA	
No	0.908 (0.466–1.770)		NA	
ALBI		0.04		0.01
Grade 1	Reference		Reference	
Grade 2	1.715 (1.008–4.152)		6.915 (1.587–19.253)	
PALBI				-
Grade 1	Reference		NA	
Grade 2	0.707 (0.397–1.260)	0.24	NA	
Grade 3	0.982 (0.562–3.213)	0.31	NA	
Ventricular drainage		0.08		-
Yes	Reference		NA	
No	0.521 (0.248–1.094)		NA	
Recurrence		0.10		-
Yes	Reference		NA	
No	0.574 (0.296–1.116)		NA	
Surgery extent				-
GTR	Reference		NA	
STR	0.698 (0.359–1.357)	0.29	NA	
Biopsy	1.112 (0.359–2.598)	0.34	NA	
Postoperative chemotherapy		0.71		-
Yes	Reference		NA	
No	1.125 (0.604–2.097)		NA	
Postoperative radiotherapy		0.84		-
Yes	Reference		NA	
No	1.068 (0.559–2.040)		NA	
SII		0.006		<0.001
Low level	Reference		Reference	
High level	2.792 (1.328–5.870)		20.502 (3.936–106.068)	
NLR		0.49		-
Low level	Reference		NA	
High level	1.438 (0.508–4.062)		NA	
MLR		0.26		-
Low level	Reference		NA	
High level	1.457 (0.756–2.809)		NA	
PNI		0.37		-
Low level	Reference		NA	
High level	0.742 (0.383–1.436)		NA	
coSII-PNI				
Score 0	Reference		Reference	
Score 1	0.45 (0.25–0.81)	0.008	0.092 (0.0091–0.93)	0.043
Score 2	0.23 (0.03–0.78)	<0.001	0.31 (0.12–0.87)	0.02

*SII, systemic immune-inflammation index; NLR, neutrophil–lymphocyte ratio; MLR, monocyte–lymphocyte ratio; PNI, prognostic nutritional index; HR, hazard ratio*.

The multivariate analysis showed that preoperative hydrocephalus (*P* < 0.001), ALBI (*P* = 0.01), and SII (*p* < 0.001). In terms of preoperative symptoms, patients having preoperative hydrocephalus (HR = 0.047; 95% CI 0.012–0.185; *p* < 0.001) increased the death risk. Patients in the high SII group (HR = 20.502; 95% CI 3.936–106.068; *p* = 0.01) had an increase in death risk compared with patients in low SII. In addition, a higher AIBL grade (HR = 6.915; 95% CI 1.587–19.253; *p* = 0.01) can significantly increase a risk of death of patient.

### Prognostic Value of the coSII-PNI in MB Undergoing Surgical Resection

**Figure 4:**
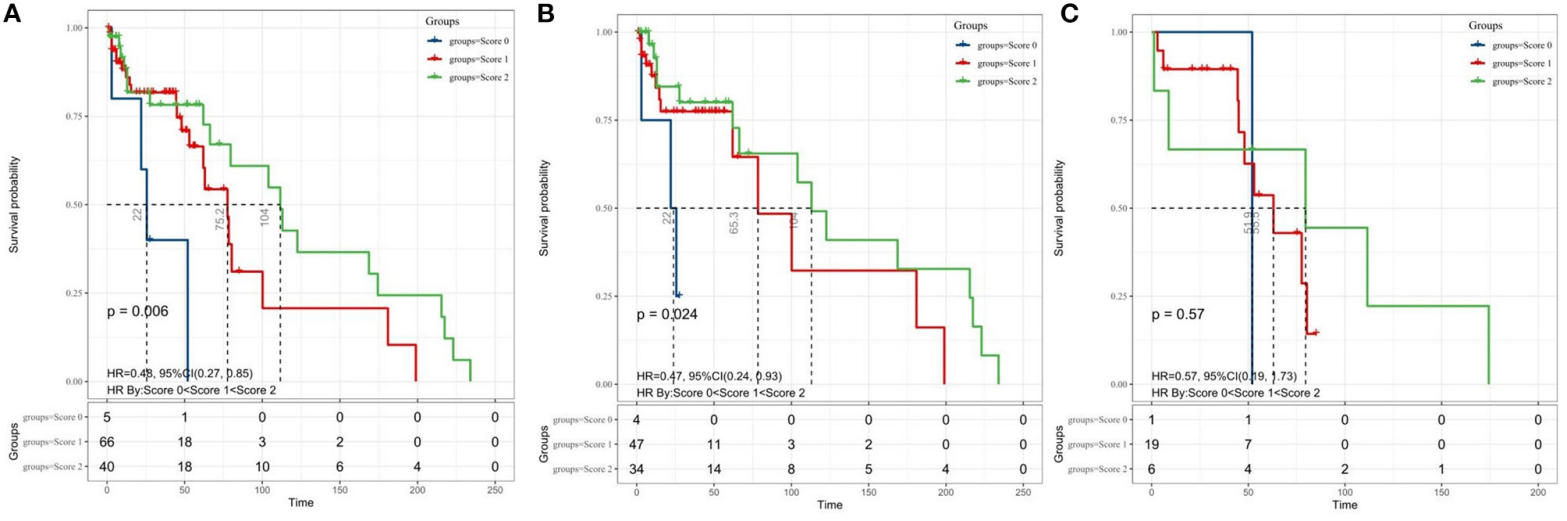
The Kaplan–Meier survival curves for OS according to the combination of SII and PNI in patients with medulloblastoma. **(A)** entire cohort; **(B)** patients aged <18 years; and **(C)** adult patients. coSII–PNI, combination of SII and PNI; SII, systemic immune-inflammation index; PNI, prognostic nutritional index.

Finally, we evaluated the prognostic value of the coSII–PNI in patients with MB undergoing surgical resection. Those with low SII and high PNI will be given a score of two; those with high SII and high PNI or low SII or low PNI score one; those with high SII and low PNI score 0. The Kaplan–Meier analysis and a log-rank test for the entire patient cohort showed that the 5-year OS for patients with coSII–PNI = 0, 1, and 2 were 0, 16.5, and 37.5%, respectively. Therefore, patients with MB were divided into three independent groups by preoperative coSII–PNI (*p* = 0.006) (Figure 4). Subgroup analysis was then performed to evaluate the prognostic value of coSII–PNI for age stratification. Under the age of 18, the highest prevalence of MB, we found coSII–PNI = 0 was inclined to have a worse OS than patients with coSII–PNI = 1 or coSII-PNI = 2 (*p* = 0.024) (Figure 4). However, no significant difference was found in adult patients (*p* = 0.57). The univariate and multivariate analyses also proved coSII–PNI was an independent prognostic factor in patients with MB (Table 3).

## Discussion

According to our best information, this study is the first attempt to specifically question the prognostic effect of the immunoinflammatory index and nutritional index on survival outcomes in newly diagnosed patients with MB undergoing surgical resection. We demonstrated that the preoperative high-level SII was linked with significantly inferior OS than low-level SII in this group of patients. Moreover, we proved that a high level of SII and higher score ALBI increased the risk of death in patients with MB. Furthermore, we introduced a new concept called coSII–PNI to predict OS in patients with MB. Therefore, in addition to recognized clinicopathological factors, namely, preoperative hydrocephalus, our results suggested a reliable and independent role of a new inexpensive and clinically relevant biomarkers SII, AIBL, and coSII–PNI for future prognosis prediction.

Several prognostic scores based on the inflammation and immunity have been developed to identify patients at high risk of relapse or death that may be difficult to identify using traditional clinicopathological indicators. However, these scores do not fully reflect the balance of host inflammatory and immune status. Therefore, we established a new indicator based on lymphocyte, neutrophil, and platelet counts, the SII index. Baseline SII can be used as an independent prognostic marker in patients with advanced pancreatic cancer with normal or elevated CA19-9 levels (9). Data obtained from the Rotterdam Study showed that higher SII at baseline was associated with a 30% increased risk of solid cancer, adjusted for age, sex, socioeconomic status, smoking status, body mass index, and type two diabetes. Absolute cumulative 10-year cancer risk increased from 9.7% in the lowest quartile of SII to 14.7% in the highest quartile of SII (*p* = 0.009) (10). The PNI is a new prognostic score calculated by multiplying albumin and lymphocyte counts to reflect inflammation and nutritional status (11). A high decrease in PNI during neoadjuvant chemotherapy predicts a poor prognosis. Maintaining nutritional status during neoadjuvant chemotherapy might lead to better treatment outcomes for the patients with breast cancer (12). Therefore, SII and PNI are prognostic indicators worthy of investigation in MB.

Studies have shown that the tumor progression, immunity, and inflammatory response have a special relationship. As a novel finding in the modern MB literatures, this study convincingly demonstrated that high levels of SII were strongly and independently associated with more inferior OS of patients with MB undergoing surgical resection. Albeit this is the first report to show an essential association between SII and the survival outcome of patients with MB, they are harmonious with previous research on other tumors of central nervous system (13–15). Therefore, a better understanding of the role of these biomarkers in MB will help explore the relationship between inflammation, immunity, and MB. However, the underlying mechanisms require further evaluation.

Patients with MB with high SII usually have elevated neutrophils, high platelets, or lymphocytopenia, indicating increased inflammation and reduced immune response. Neutrophils have been reported to be involved in the tumor formation and progression through various mechanisms, including vascular endothelial growth factor-mediated angiogenesis and tumor immunosuppression (16). Study of Karajannis et al. already proved patients with MB have a higher neutrophil count suggesting patients already had tumor-induced systemic immunosuppression at the time of diagnosis (17). On the other hand, platelets play an active role in all steps of tumorigenesis, including tumor growth, tumor cell exosmosis, and metastasis. In addition, thrombocytosis in patients with cancer is associated with poor survival. Due to many particles and exosomes secreted, platelets can well coordinate local and distant tumor-host crosstalk (18). Expression of platelet-derived growth factors was also common in gliomas (19). Hambardzumyan et al. study demonstrated platelet-derived growth factor-beta is a potent inflammatory driver in pediatric MB (19). Lymphocytes reflect host immune status and mediate host immune response to cancer. Meanwhile, lymphocytes have an antitumor effect by inhibiting proliferation and invasion of cancer cells and promoting apoptosis of tumor cells. Tumor-infiltrating lymphocytes play an essential role in mediating responses to chemotherapy and improving cancer clinical outcomes (20). What is more, systemic inflammation may increase neutrophils count and decrease lymphocyte count, which may lead to a reduction in cell-mediated cytotoxic immune response, leading to treatment failure (21). To confirm the difference of the inflammatory and the immune response status in the molecular subgroups, Zhang et al. showed that the preoperative levels of NLR in the group three MB were significantly higher than those in the WNT group, and the preoperative high NLR was negatively correlated with OS in group three (*p* = 0.032) and group four (*p* = 0.027) (22). Therefore, anti-inflammatory therapy or immunotherapy may be an effective treatment strategy for patients with MB with high SII. These studies may help shed light on why patients with high SII have worse outcomes and suggest the potential for anti-inflammatory therapy in patients with MB, although further research is needed to verify this hypothesis.

Index has been extensively studied in other CNS tumors. The recent studies suggest that NLR may be a novel biomarker for the prognostic stratification of R-GBMS treated with BEVIRI (23). Another study conducted by Dr. Erkan Topkan (24) showed that preconditioning pretreatment systemic immune response index (SIRI) is a new, reliable, and independent predictor of prognosis for newly diagnosed patients with GBM intending to undergo postoperative Stupp regiments. As a tumor of the central nervous system, the prognostic prediction of MB by the cerebrospinal fluid (CSF) is progressing rapidly. For the common tumor markers seen in the blood, the CSF tends to show a different performance. This study found that circulating tumor DNA (ctDNA) is abundant in CSF but almost absent in plasma, and longitudinal analysis of ctDNA in CSF is useful in studying the characteristics of minimal residual disease, genomic evolution, and the recurrent tumors in MB (25). However, no prognostic value was found for immunoinflammatory markers in CSF. In fact, researchers have found that proteomic analysis identified typical tumor markers, including FSTL5, ART3, and FMOD, and revealed the prevalence of anti-inflammatory and tumor-promoting proteins that are characteristic of myeloid cells in CSF from patients with MB (26). This also provides some theoretical basis for the application of the immune inflammation index in MB in the future.

Albumin–Bilirubin score, a well-designed marker composed of bilirubin and albumin, can be used as an assessment tool for hepatic function. Albumin can be a multifunctional protein carrier system for tumor therapy. As a carrier, it can provide tumor specificity, reduce drug-related toxicity, and maintain the therapeutic concentration of active parts such as drugs, genes, peptides, and proteins (27). Albumin nanoparticles for glutathione-responsive release of cisplatin can be used in the treatment of MB and this dramatically increases the efficiency of cisplatin crossing the blood–brain barrier (28). Bilirubin, a by-product of the breakdown of hemoglobin, is said to be an antioxidant and is thought to protect against cancer (29). Moderately elevated serum bilirubin levels are associated with a lower incidence of lung cancer, especially among smokers. It is not clear whether these relationships reflect antioxidant properties or residual confusion of cancer (30). As a nutritional index to evaluate liver function, it is often used to evaluate hepatocellular carcinoma (HCC) tolerance of patients to treatment (31). A higher ALBI grade predicted a poorer prognosis for HCC (32). Our study was the first one in tumors other than patients with HCC to illustrate significant connections between the ALBI grade and survival outcomes. The specific mechanism of the liver metabolic nutrition and prognosis of MB deserves further investigation.

Prognostic nutritional index is considered to be a potent negative prognostic factor in many cancers (33, 34). However, no previous studies had characterized prognostic value of PNI in MB. Similarly, PNI was not an independent prognostic factor in our study. We considered that the deviation might be caused by insufficient sample size and retrospective study. Besides, the findings suggested that early aggressive nutritional interventions need to be considered to prevent nutritional decline during MB treatment (35). Therefore, we utilized a combinative analysis of SII and PNI to predict the OS of patients with MB. Our data showed that patients with high PNI and low SII had the best prognosis, while patients with low PNI and high SII had the worst prognosis. In age <18 years patients who are high-risk groups of MB, coSII–PNI was significantly correlated with OS (*P* = 0.0024). All the aforementioned results suggest that we cannot ignore the predictive role of PNI in MB, which needs to be validated in large prospective clinical trials.

This study has some certain hindrances. First, this is a single-institution retrospective cohort analysis in a relatively small cohort. Therefore, our findings should be interpreted with considerable caution until consistent results of prospective hypotheses with the large-scale confirmatory studies are possible. Second, tumor-related variables such as molecular subgroups and local/systemic reactive proinflammatory cytokine/chemokine levels did not allow us to perform SII group analysis based on these biomarkers. Third, several reported evaluations of inflammatory markers, including C-reactive protein, Glasgow prognostic score, interleukin, and tumor necrosis factor, were not included in this study (36). Last but not the least, unforeseeable differences between the salvage maneuvers at recurrence may have altered the outcomes in favor of one group in an unintentional manner. Therefore, more extensive prospective studies, especially confirmatory studies and Hematological indexes measured at multiple times, are needed in the future to confirm our preliminary results.

## Conclusion

The present endeavor exploring the prognostic significance of immune-nutritional index on survival results of the patients with MB undergoing surgical resection setting exhibited that the reproducibly measurable, cost-effective, and easily calculated SII, AIBL, and coSII–PNI were predictors of OS in patients with MB. If consistent with the results of future large-scale studies, we rationally believe that future well-designed studies addressing these questions may provide valuable insights into the mechanistic relationship of these biomarkers in patients with MB.

## Data Availability Statement

The original contributions presented in the study are included in the article/supplementary material, further inquiries can be directed to the corresponding authors.

## Ethics Statement

The studies involving human participants were reviewed and approved by Institutional Review Board of Sun Yat-sen University Cancer Center. Written informed consent to participate in this study was provided by the participants' legal guardian/next of kin.

## Author Contributions

SZ and ZCheng conducted data analysis and drafted the manuscript. YH, ZChen, and JZ participated in the data collection. JW, YC, and FL participated in the design of the study. All authors read and approved the final manuscript.

## Conflict of Interest

The authors declare that the research was conducted in the absence of any commercial or financial relationships that could be construed as a potential conflict of interest.

## Publisher's Note

All claims expressed in this article are solely those of the authors and do not necessarily represent those of their affiliated organizations, or those of the publisher, the editors and the reviewers. Any product that may be evaluated in this article, or claim that may be made by its manufacturer, is not guaranteed or endorsed by the publisher.
